# Preoperative neutrophil percentage-to-albumin ratio as a postoperative AKI predictor in non-cardiac surgery: a retrospective cohort secondary analysis

**DOI:** 10.1038/s41598-025-12949-w

**Published:** 2025-07-31

**Authors:** Lei Lei, Yuling Liang, Jingyan Chen, Tianjiao Cui, Junxuan Fang, Lingyan Fei, Wenjian Lin, Chun Tang, Shan Jiang, Xiaohua Wang

**Affiliations:** Department of Nephrology, Center of Kidney and Urology, The Seventh Affiliated Hospital, Sun Yat-sen University, Shenzhen, China

**Keywords:** Acute kidney injury, Neutrophil Percentage-to-Albumin ratio, Non-cardiac surgery, Predictor, Diseases, Medical research, Nephrology, Risk factors

## Abstract

Acute kidney injury (AKI) is a critical postoperative complication in non-cardiac surgery patients, significantly impacting patient outcomes. The neutrophil percentage-to-albumin ratio (NPAR) is a promising inflammatory biomarker for predicting AKI. However, it is still unclear whether NPAR could be used as a predictor of postoperative AKI in Non-Cardiac Surgical Patients. Univariate and multivariable logistic regression analyses were conducted to assess the predictive value of NPAR for postoperative AKI, controlling for potential confounders. A total of 3041 patients were considered for the analysis after excluding those with preoperative infections and chronic kidney disease. The area under the receiver operating characteristic (ROC) curve for NPAR was 0.723, indicating moderate predictive capability for postoperative AKI. The optimal threshold for NPAR was 5.310, with a specificity of 0.640 and a sensitivity of 0.729. Multivariable regression analysis revealed that NPAR was significantly associated with postoperative AKI risk (adjusted odds ratio 1.093, 95% CI 1.072–1.116, *P* < 0.001), independent of other clinical factors. Preoperative NPAR is a significant predictor of postoperative AKI in non-cardiac surgical patients under general anesthesia and could be a valuable biomarker for identifying non-cardiac surgical patients at high-risk of AKI.

**Supplementary Information:**

The online version contains supplementary material available at 10.1038/s41598-025-12949-w.

## Introduction

Postoperative Acute Kidney Injury (AKI) is a prevalent and notable clinical complication that emerges after surgery, characterized by a rapid deterioration of renal function within a brief period^1,2^. Globally, the incidence of postoperative AKI is considerable, with a particularly disconcerting prevalence in intensive care units (ICUs), affecting 13.5–55.9% of patients undergoing major surgeries^3–5^. The multifactorial etiology of postoperative AKI includes pre-renal conditions, such as intraoperative hypovolemia and reduced cardiac output^6^; intrinsic renal factors, including ischemia-reperfusion injury and acute tubular necrosis^7^; and post-renal causes associated with urinary tract obstruction^8,9^.

Postoperative AKI is significantly associated with prolonged hospital stays, elevated mortality rates, and increased healthcare costs, imposing a substantial burden on both patients and healthcare systems^5^. The pathogenesis of AKI is complex, involving a range of pathophysiological processes. These include alterations in renal hemodynamics, direct tubular cell damage, inflammation, oxidative stress, and cell death^10–12^. Renal tubular injury constitutes a central element of AKI pathogenesis, with mechanisms such as ischemia-reperfusion injury, toxic insults from anesthetics or medications, and inflammatory responses triggered by surgery or infection^11,13,14^. Specifically, inflammation is a crucial driver in the progression of tubular injury, leading to additional cell damage, renal vasoconstriction, and interstitial fibrosis, which can contribute to the severity and persistence of postoperative AKI^15,16^.

The neutrophil percentage-to-albumin ratio (NPAR) has emerged as a novel inflammatory biomarker, garnering significant interest in recent years^17–19^. Neutrophils, which are key players in inflammation, increase in number during inflammatory states, while albumin levels typically decrease^20,21^. Studies have implicated NPAR in the pathogenesis and prognosis of various conditions, including cancers^22,23^, metabolic syndrome^24^and chronic kidney disease (CKD)^25^. However, the relationship between NPAR and the development of postoperative AKI following surgery remains largely unexplored. In the present study, we aim to investigate the correlation between NPAR and postoperative AKI to identify high-risk individuals and enhance diagnostic accuracy by discovering a novel biomarker for risk stratification and diagnosis.

## Methods

### Study design and data source

This study utilizes the open data set from the research article *Preoperative Neutrophil-Lymphocyte Ratio for predicting surgery-related acute kidney injury in non-cardiac surgery patients under general anaesthesia: A retrospective cohort study* published in 2022 written by Tang et al.^26^. The dataset comprises clinical information from patients who underwent general anesthesia for non-cardiac surgery at the Third Xiangya Hospital of Central South University (January 2012 to December 2016) and the Second Xiangya Hospital of Central South University (January 2016 to December 2016). The dataset can be accessed through the Dataverse platform with the DOI 10.1371/journal.pone.0270066.

### Definitions

Postoperative AKI was identified based on the Kidney Disease: Improving Global Outcomes (KDIGO) creatinine criteria, which includes either a serum creatinine rise of ≥ 0.3 mg/dL within a 48-hour period or a serum creatinine elevation to at least 1.5 times the baseline level within the first seven days postoperatively^27^. The baseline serum creatinine value was determined as the minimum value recorded in the week preceding surgery. The NPAR^17^ was calculated using the following formula:

NPAR=(neutrophil percentage (%)×100) / albumin (g/dL).This formula quantifies the ratio of neutrophils to serum albumin levels, with the neutrophil percentage expressed as a proportion of the total white blood cell count and multiplied by 100 to normalize the value, while albumin is measured in grams per deciliter.

### Statistical analyses

To assess the relationship between individual variables and the occurrence of postoperative AKI, we performed univariate analysis. Continuous variables with nonnormal distribution were compared using the Mann-Whitney U test, while categorical variables were assessed using the Chi-square test or Fisher’s exact test, as appropriate. All tests were two-tailed, and *P* < 0.05 was considered statistically significant. The predictive performance of preoperative NPAR for postoperative AKI was evaluated using receiver operating characteristic (ROC) analysis. The area under the ROC curve (AUC) was calculated to quantify the ability of NPAR to discriminate AKI and non-AKI. The optimal threshold for NPAR was determined using the Youden Index (Sensitivity + Specificity − 1) from the ROC curve analysis, which maximizes the combined diagnostic performance of sensitivity and specificity. Multivariable regression analysis was conducted to investigate the independent association between NPAR and postoperative AKI while controlling for potential confounders. Variables with *P* < 0.1 in univariate analysis were included in the initial multivariable model. Prior to variable selection, covariates in the regression model were screened for multicollinearity using variance inflation factor (VIF), and variables with VIF > 10 were excluded from the model. A backward elimination process was then applied to refine the model, retaining variables with *P* < 0.05. Statistical analyses were performed using IBM SPSS software (version 22.0) for univariate analysis and R software (version 2.12.0) for ROC analysis and multivariable regression modeling.

### Ethical approval

This study was conducted in accordance with the ethical standards of the Declaration of Helsinki and approved by the Ethics Committee of the Third Xiangya Hospital, Central South University (Approval No. 2017-S214). Due to the observational nature of the research and the use of anonymized clinical data, the requirement for informed consent from participants was waived by the Ethics Committee of the Third Xiangya Hospital, Central South University. This exemption aligns with Article 32 of the Ethical Review Measures for Life Sciences and Medical Research Involving Humans (issued by the National Health Commission of China, 2023), which stipulates that studies utilizing de-identified data without involving sensitive personal information or commercial interests may be exempt from ethical review. All methods were performed in compliance with relevant national regulations and institutional guidelines.

## Results

### Study cohort and data collection

**Fig. 1:**
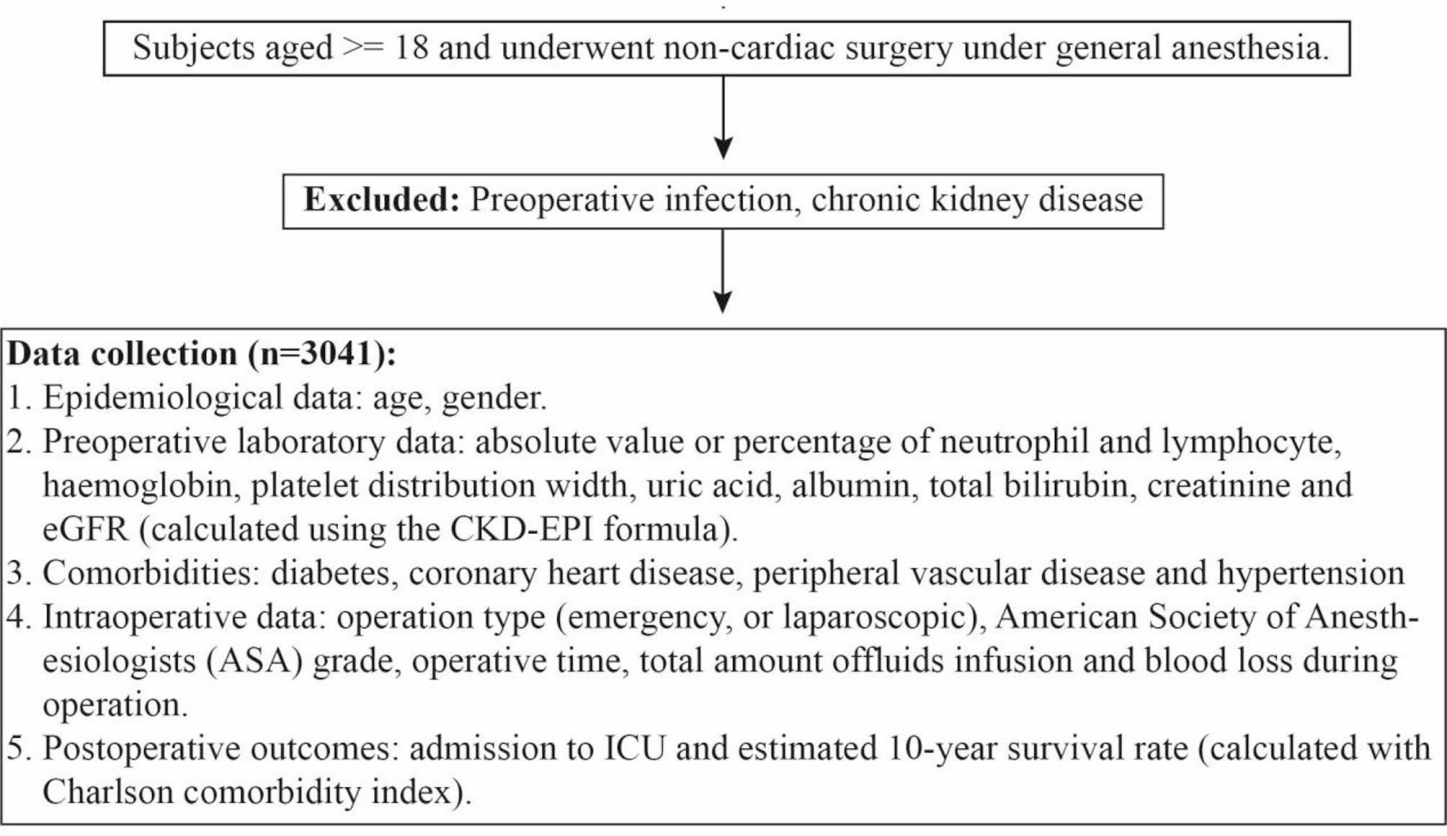
Flow diagram of the study cohort and data collection. CKD-EPI, Chronic Kidney Disease epidemiology collaboration.

The study included subjects aged 18 years or older who underwent non-cardiac surgery under general anesthesia. A total of 3041 patients were considered for the analysis. Patients with preoperative infections and those with chronic kidney disease were excluded from the study to ensure the cohort homogeneity and to focus on the effects of non-cardiac surgery on AKI risk. Data collection encompassed epidemiological, preoperative laboratory, comorbidity, intraoperative, and postoperative outcomes data (Fig. 1.).

### Association of preoperative or postoperative parameters with NPAR

Table 1 illustrated the evaluation of various preoperative or postoperative parameters and their correlation with the level of NPAR. Data from 3041 patients were stratified into low, middle, and high tertiles based on NPAR, with corresponding sample sizes of *N* = 1014, *N* = 1013, and *N* = 1014, respectively (Table 1). The mean NPAR values were 1.81 (SD = 0.47, Table 1), 4.07 (SD = 0.96, Table 1), and 11.17 (SD = 6.74, Table 1) across the low, middle, and high tertiles, respectively, with highly significant differences between groups (*P* < 0.001, Table 1).

**Table 1:** Clinical characteristics and outcomes across NPAR tertiles.

Parameter		Low NPAR (*N* = 1014)	Middle NPAR (*N* = 1013)	High NPAR (*N* = 1014)	*P*-value
NPAR		1.81 (0.47)	4.07 (0.96)	11.17 (6.74)	***
WBC (×10^9^/L)		5.74 (1.40)	8.62 (2.33)	14.57 (5.08)	***
Neutrophils (×10^9^/L)		3.38 (1.01)	6.61 (2.05)	12.80 (4.78)	***
Lymphocytes (×10^9^/L)		1.75 (0.73)	1.39 (0.72)	1.07 (0.68)	***
RBC (×10^12^/L)		4.45 (0.57)	4.18 (0.64)	3.70 (0.78)	***
Hb (g/L)		129.17 (18.83)	122.03 (21.25)	109.72 (24.13)	***
PLT (×10^9^/L)		224.06 (77.81)	238.50 (98.13)	218.91 (112.02)	***
NLR		0.23 (0.16)	0.54 (0.20)	0.85 (0.21)	***
Alb (g/L)		42.11 (4.02)	39.01 (5.21)	33.96 (7.03)	***
Total protein (g/L)		68.33 (6.20)	65.25 (8.00)	58.03 (10.66)	***
Glb (g/L)		26.22 (4.48)	26.33 (5.35)	24.07 (5.90)	***
ALT (g/L)		32.22 (45.19)	36.09 (69.76)	51.28 (116.53)	***
TBIL (µmol/L)		16.19 (14.01)	21.41 (37.33)	21.23 (27.39)	***
DBIL (µmol/L)		6.15 (9.52)	10.13 (26.79)	10.58 (20.46)	***
Total bile acids (µmol/L)		5.17 (10.37)	6.60 (23.65)	5.83 (22.53)	0.266
PNI		50.84 (5.73)	45.98 (6.90)	39.29 (8.06)	***
ALBI		-0.94 (0.53)	-0.60 (0.76)	-0.12 (0.84)	***
Creatinine (µmol/L)		65.89 (15.58)	64.67 (16.84)	62.95 (18.17)	***
Uric Acid (µmol/L)		275.83 (94.21)	244.08 (95.47)	211.11 (100.43)	***
eGFR (mL/min/1.73m^2^)		102.53 (20.23)	103.16 (18.93)	105.08 (22.12)	0.014
Male n (%)		493 (48.62%)	543 (53.60%)	559 (55.13%)	0.009
Age		51.62 (14.36)	52.68 (14.11)	51.86 (15.72)	0.24
Height (in)		51.10 (75.84)	44.60 (72.74)	40.64 (70.49)	0.005
Weight (kg)		56.12 (18.09)	53.62 (18.21)	51.23 (22.73)	***
Emergency indicator n (%)		57 (5.62%)	211 (20.83%)	352 (34.71%)	***
Laparoscopy n (%)		317 (31.26%)	237 (23.40%)	151 (14.89%)	***
Surgery duration		3.28 (1.79)	3.26 (1.70)	3.43 (1.82)	0.069
Volume of blood loss (mL)		361.42 (438.38)	403.21 (496.47)	619.01 (884.65)	***
Volume of fluid intake (mL)		3045.80 (1229.04)	2943.96 (1199.06)	3084.50 (1305.31)	0.032
Volume of fluid output (mL)		852.33 (756.93)	774.15 (601.80)	860.81 (644.97)	0.006
Anesthesia duration (hours)		4.28 (1.99)	4.33 (1.90)	4.53 (2.01)	0.011
Operation scale n (%)	1	8 (0.79%)	6 (0.59%)	12 (1.18%)	0.319
	2	266 (26.23%)	289 (28.53%)	253 (24.95%)	0.319
	3	700 (69.03%)	685 (67.62%)	703 (69.33%)	0.319
	4	40 (3.94%)	33 (3.26%)	46 (4.54%)	0.319
ASA grade n (%)	1	86 (8.48%)	55 (5.43%)	29 (2.86%)	***
	2	705 (69.53%)	598 (59.03%)	460 (45.36%)	***
	3	215 (21.20%)	322 (31.79%)	414 (40.83%)	***
	4	7 (0.69%)	38 (3.75%)	105 (10.36%)	***
	5	1 (0.10%)	0 (0.00%)	6 (0.59%)	***
Pre-DM n (%)		169 (16.67%)	195 (19.25%)	197 (19.43%)	0.2
Pre-CHD n (%)		52 (5.13%)	75 (7.40%)	102 (10.06%)	***
Pre-PVD n (%)		127 (12.52%)	83 (8.19%)	88 (8.68%)	0.002
Pre-HF n (%)		3 (0.30%)	0 (0.00%)	1 (0.10%)	0.174
Pre-HTN n (%)		461 (45.46%)	439 (43.34%)	397 (39.15%)	0.014
Estimated 10-year survival rate		0.88 (0.17)	0.86 (0.19)	0.85 (0.31)	***
Postoperative AKI n (%)		12 (1.18%)	41 (4.05%)	98 (9.66%)	***

The table presents data using two primary formats: mean values accompanied by their standard deviation (SD) for continuous variables, and the count of occurrences along with the percentage (%) represents for categorical variables. For statistical significance, *** denotes a *P* < 0.001. WBC, White Blood cell; Hb, hemoglobin; PLT, platelets; Alb, albumin; Glb, globulin; ALT, alanine aminotransferase; TBIL, total bilirubin; DBIL, direct bilirubin; PNI, preoperative nutrition index = serum albumin (g/L) + 0.005×lymphocyte count (cells/mm^3^); ALBI, Albumin-bilirubin index = $\frac{{\ln \left( {{\text{total bilirubin}}\left( {\mu {\text{mol/L}}} \right)} \right) \times 0.66+\ln \left( {{\text{albumin}}\left( {{\text{g}}/{\text{L}}} \right) \times \left( {0.085} \right)} \right)}}{{10}}$;eGFR, estimated glomerular filtration rate; Pre-DM, preoperative diabetes mellitus; Pre-CHD, preoperative coronary heart disease; Pre-PVD, preoperative peripheral vascular disease; Pre-HF, preoperative heart failure; Pre-HTN, preoperative hypertension; AKI, acute kidney injury; ASA, American Society of Anesthesiologists (ASA grade 1: A healthy patient without systemic disease, ASA grade 2: A patient with mild systemic disease, ASA grade 3: A patient with severe systemic disease, ASA grade 4: A patient with severe systemic disease that is a constant threat to life, ASA grade 5: A moribund patient who is not expected to survive without surgery).

White blood cell (WBC) and neutrophil counts increased with higher NPAR tertiles, while lymphocyte counts decreased in the highest NPAR tertile. Red blood cell (RBC) count decreased significantly from 4.45 ± 0.57 to 3.70 ± 0.78 (10¹²/L), while neutrophil-to-lymphocyte ratio (NLR) increased nearly fourfold from 0.23 to 0.85 (*P* < 0.001, Table 1). Hemoglobin (Hb) levels declined progressively from 129.17 ± 18.83 g/L in low tertile to 109.72 ± 24.13 g/L in high tertile, and platelet (PLT) levels declined, though middle tertile showed a transient increase (238.50 ± 98.109/L, Table 1). Total protein exhibited a marked decline from 68.33 ± 6.20 to 58.03 ± 10.66 g/L (*P* < 0.001, Table 1) and globulin (Glb) levels decreased from 26.22 ± 4.48 to 24.07 ± 5.90 g/L (*P* < 0.001, Table 1). Albumin (Alb) levels demonstrated a stepwise decline across NPAR tertiles paralleled by a progressive elevation in alanine aminotransferase (ALT) levels, with both trends reaching statistical significance (*P* < 0.001, Table 1). Total bilirubin (TBIL) and direct bilirubin (DBIL) levels were also significantly associated with higher NPAR tertiles (*P* < 0.001, Table 1). Nutritional parameters deteriorated progressively, with preoperative nutrition index (PNI) decreasing from 50.84 ± 5.73 to 39.29 ± 8.06 and albumin-bilirubin index (ALBI) worsening from − 0.94 ± 0.53 to -0.12 ± 0.84 (*P* < 0.001, Table 1). Creatinine and uric acid levels showed a decreasing trend (*P* < 0.001, Table 1), while the estimated glomerular filtration rate (eGFR) showed a modest elevation across tertiles (*P* = 0.014, Table 1). Body weight was significantly reduced in the highest NPAR tertile (*P* < 0.001, Table 1). Age and height showed no significant differences across NPAR tertiles, while surgery duration showed borderline significance (*P* = 0.069, Table 1) and anesthesia duration was significantly prolonged (4.28 ± 1.99 to 4.53 ± 2.01 h, *P* = 0.011, Table 1). Intraoperative fluid intake showed U-shaped variation (*P* = 0.032, Table 1), while output displayed nonlinear changes (*P* = 0.006, Table 1). Blood loss escalated dramatically from 361.42 ± 438.38 ml to 619.01 ± 884.65 ml (*P* < 0.001, Table 1). The prevalence of coronary heart disease (CHD) demonstrated a significant stepwise increase across NPAR tertiles ( *P* < 0.001, Table 1). Conversely, both peripheral vascular disease (PVD) and hypertension (HTN) showed inverse associations with NPAR levels, with PVD decreasing from 12.52 to 8.68% (*P* = 0.002, Table 1) and HTN declining from 45.46 to 39.15% (*P* = 0.014, Table 1). Notably, neither diabetes mellitus (DM) nor preoperative heart failure (HF) exhibited statistically significant associations with NPAR tertiles. The proportion of ASA III-V patients demonstrated a progressive increase across NPAR tertiles, paralleled by escalating postoperative AKI incidence (*P* < 0.001, Table 1). These findings highlight the importance of a high NPAR as a robust predictor of increased risk for postoperative and other adverse outcomes in patients undergoing non-cardiac surgery under general anesthesia.

### Univariate logistic regression analysis for risk factors of postoperative AKI

We conducted univariate logistic regression analysis to identify potential risk factors associated with the development of AKI following non-cardiac surgery under general anesthesia (Table 2). A total of 3041 patients were included in the analysis, with 151 cases (4.96%, Table 2) of postoperative AKI.

**Table 2:** Univariate logistic regression analysis for AKI.

Parameter	*N* (%) /Mean ± SD	OR (95% CI)	*P*-value
Male (n %)	1595 (52.45%)	1.18 (0.85, 1.64)	0.3327
Weight (kg)	53.66 ± 19.89	0.98 (0.98, 0.99)	< 0.001
Height (in)	45.45 ± 73.16	1.00 (0.99, 1.00)	0.0226
Pre-HTN (n %)	1297 (42.65%)	1.55 (1.12, 2.15)	0.0089
Pre-CHD (n %)	229 (7.53%)	1.72 (1.03, 2.87)	0.0382
Pre-DM (n %)	561 (18.45%)	1.47 (1.00, 2.15)	0.0503
Pre-PVD (n %)	298 (9.80%)	1.71 (1.08, 2.71)	0.0227
Pre-HF (n %)	4 (0.13%)	6.42 (0.66, 62.05)	0.1084
Emergency indicator	620 (20.39%)	4.37 (3.13, 6.09)	< 0.001
Laparoscopy (n %)	705 (23.18%)	0.43 (0.26, 0.72)	0.0011
Surgery Duration (hours)	3.32 ± 1.77	1.14 (1.05, 1.23)	0.0016
Volume of fluid intake (mL)	3024.78 ± 1246.29	1.00 (1.00, 1.00)	0.0429
Volume of fluid output (mL)	829.11 ± 672.03	1.00 (1.00, 1.00)	0.0072
Volume of blood loss (mL)	461.23 ± 647.78	1.00 (1.00, 1.00)	< 0.001
Anesthesia duration (hours)	4.38 ± 1.97	1.13 (1.05, 1.22)	0.0007
ASA grade	2.36 ± 0.67	3.12 (2.50, 3.90)	< 0.001
Hb (g/L)	120.30 ± 22.96	0.98 (0.98, 0.99)	< 0.001
PLT (×10^9^/L)	227.15 ± 97.33	1.00 (0.99, 1.00)	0.0028
ALT (g/L)	39.87 ± 83.02	1.00 (1.00, 1.00)	0.1109
Uric acid (µmol/L)	243.67 ± 100.26	1.00 (0.99, 1.00)	< 0.001
Creatinine (µmol/L)	64.51 ± 16.93	0.99 (0.98, 1.00)	0.1374
NPAR	5.69 ± 5.61	1.09 (1.07, 1.12)	< 0.001

Data are shown as number (%) or mean ± SD. OR (odds ratio), CI (confidence interval).

Patients with higher body weight exhibited a significantly reduced risk of postoperative AKI, with an odds ratio (OR) of 0.98 per unit increase (95% CI: 0.98–0.99, *P* < 0.001, Table 2). Preoperative hypertension, coronary artery disease, and peripheral vascular disease were significantly associated with an increased risk of postoperative AKI (*P* < 0.05, Table 2). In contrast, preoperative diabetes and heart failure were associated with a non-significant increase in the risk of postoperative AKI. Prolonged operative time and anesthesia duration were significantly correlated with an elevated risk of postoperative AKI (*P* < 0.01, Table 2). There was a significant association between greater intraoperative blood loss and postoperative AKI (OR: 1.00,95% CI: 1.00–1.00, *P* < 0.001, Table 2). A higher ASA physical status classification was significantly associated with an increased risk of postoperative AKI (OR: 3.12 per grade increase, 95% CI: 2.50–3.90, *P* < 0.001, Table 2). Hemoglobin levels, platelet counts, and uric acid levels were significantly associated with the risk of AKI, whereas creatinine levels did not show a significant association with postoperative AKI risk. NPAR was significantly associated with an increased risk of postoperative AKI (OR: 1.09, 95% CI: 1.07–1.12, *P* < 0.001, Table 2).

The univariate logistic regression analysis revealed several significant risk factors for postoperative AKI, including male sex, decreased body weight, preoperative hypertension, peripheral vascular disease, extended duration of surgery and anesthesia, increased blood loss, higher ASA grade, and specific laboratory parameters such as hemoglobin levels, platelet counts, uric acid levels, and NPAR.

### Higher NPAR levels indicate a greater risk of postoperative AKI

**Fig. 2:**
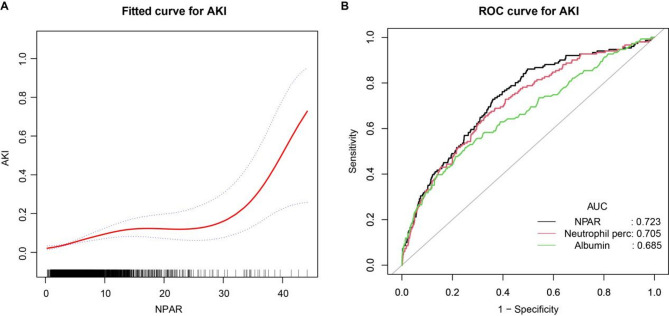
NPAR Analysis with Fitted Curve and ROC Curve for Predicting Postoperative AKI. (A) Relationship between preoperative NPAR values and the risk of postoperative AKI. The horizontal axis represents the preoperative NPAR value, and the vertical axis represents the estimated probability of developing postoperative AKI. The solid red line denotes the fitted logistic regression curve, illustrating the trend of increasing AKI risk with higher NPAR levels. The dotted red lines surrounding the fitted curve represent the 95% confidence intervals, indicating the range of uncertainty around the estimated probability. (B) Receiver operating characteristic (ROC) curves comparing the predictive performance of NPAR, neutrophil percentage, and albumin for postoperative AKI. The diagonal gray line (AUC = 0.5) serves as a reference for a non-informative predictor. Each colored curve corresponds to a specific biomarker: the blue curve represents NPAR (AUC = 0.723, 95% CI: 0.687–0.769), the orange curve represents neutrophil percentage (AUC = 0.705, 95% CI: 0.659–0.747), and the green curve represents albumin (AUC = 0.685, 95% CI: 0.639–0.722). The area under the curve (AUC) values are annotated adjacent to their respective curves to highlight their discriminative power.

Figure 2.A illustrates the association between increased NPAR and a heightened risk of developing postoperative AKI, with the risk significantly rising when the NPAR exceeds 30. A comprehensive analysis of NPAR’s predictive value for AKI following non-cardiac surgery yielded an AUC of 0.723 (95% CI: 0.687–0.769, Fig. 2.B, Table 3), indicating moderate predictive capability. The optimal NPAR threshold was 5.310, with a specificity of 0.640 and a sensitivity of 0.729 (Table 3). The diagnostic OR for NPAR was 4.773, with a positive predictive value (Positive-pv) of 0.096 and a negative predictive value (Negative-pv) of 0.978 (Table 3).

**Table 3:** ROC analysis for predictors of AKI.

Predictor	NPAR	Neutrophil	Albumin
Total Cases (AKI/Non AKI)	2890 (151/2739)	2890 (151/2739)	2890 (151/2739)
AUC (95% CI)	0.726(0.687–0.769)	0.705(0.659–0.747)	0.685 (0.639–0.722)
Best Threshold	5.310	2.149	35.15
Specificity	0.640	0.669	0.748
Sensitivity	0.729	0.656	0.517
Accuracy	0.645	0.669	0.737
Positive-LR	2.024	1.982	2.053
Negative-LR	0.424	0.515	0.646
Diagnose-OR	4.773	3.852	3.179
N-for-Diagnose	2.713	3.079	3.774
Positive-pv	0.096	0.094	0.097
Negative-pv	0.978	0.974	0.967

Positive-LR, positive likelihood ratio; Negative-LR, negative likelihood ratio; Diagnosis OR, diagnostic odds ratio; N for diagnose, number needed to diagnose; Positive pv, positive predictive value; Negative-pv, negative predictive value.

The neutrophil percentage also demonstrated moderate predictive power for postoperative AKI, with an AUC of 0.705 (95% CI: 0.659–0.747, Fig. 2B, Table 3). The best threshold for neutrophil percentage was 2.149, providing a specificity of 0.669 and a sensitivity of 0.656 (Table 3). The diagnostic OR was 3.852, with a Positive-pv of 0.094 and a Negative-pv of 0.974 (Table 3).

Albumin had the least predictive power among the three markers, with an AUC of 0.685 (95% CI: 0.639–0.722, Fig. 2B, Table 3). The optimal threshold for albumin was 35.15, offering a specificity of 0.748 and a sensitivity of 0.517 (Table 3). The diagnostic OR for albumin was 3.179, with a Positive-pv of 0.097 and a Negative-pv of 0.967 (Table 3). ROC curve analysis (Fig. 2.B, Table 3) indicates that NPAR outperforms both neutrophil percentage and albumin in predicting postoperative AKI.

### Multivariable regression analysis of clinical variables and postoperative AKI

**Fig. 3:**
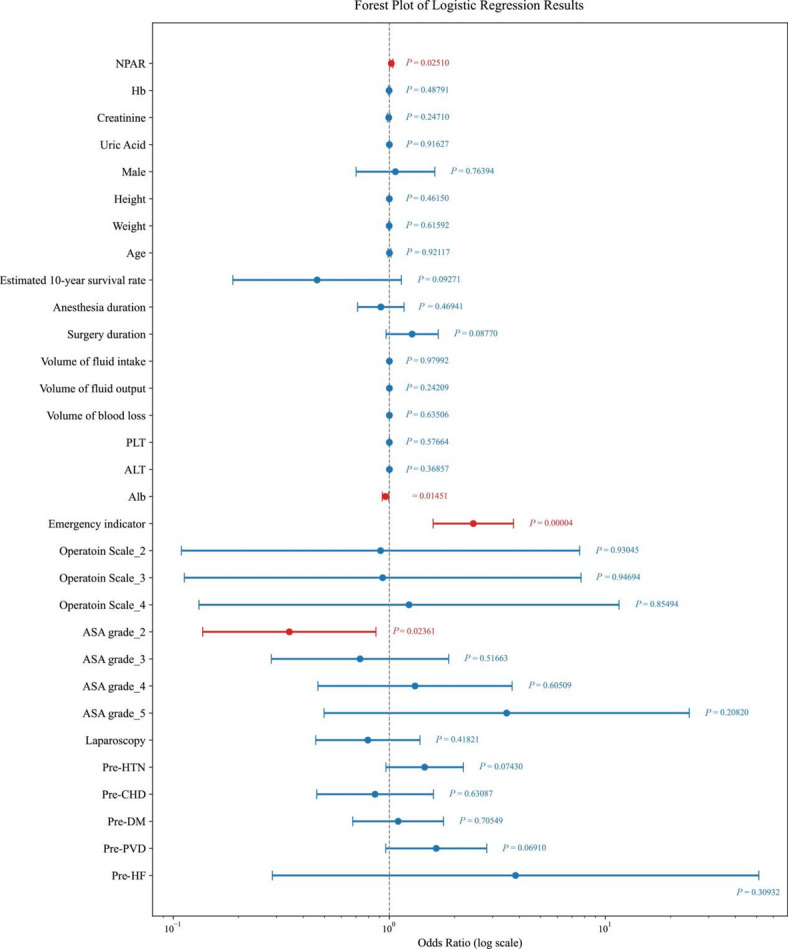
Forest plot of multivariable regression analysis showing the association between clinical variables and the outcome. The forest plot illustrates the odds ratios (ORs) and 95% confidence intervals (CIs) for each variable included in the multivariate logistic regression model predicting postoperative AKI. Variables with CIs that do not cross the null value (OR = 1) are considered statistically significant. Red dots and lines indicate significant predictors (*P* < 0.05), while blue dots and lines represent non-significant predictors. The horizontal lines represent the 95% CIs, and the vertical dashed line represents the null value (OR = 1). *P*-values are provided for each variable. Hb, hemoglobin; PLT, platelets; Alb, albumin; ALT, alanine aminotransferase; Pre-DM, preoperative diabetes mellitus; Pre-CHD, preoperative coronary heart disease; Pre-PVD, preoperative peripheral vascular disease; Pre-HF, preoperative heart failure; Pre-HTN, preoperative hypertension.

A multivariate logistic regression analysis was conducted to evaluate the association between various preoperative and intraoperative factors and the occurrence of postoperative AKI. The results are presented in a forest plot (Fig. 3), which revealed several significant predictors of AKI. Notably, NPAR (OR = 1.0203, 95% CI: 1.0025 to 1.0385, *P* = 0.025, Fig. 3, Supplementary Table S1), Alb levels (OR = 0.95867, 95% CI: 0.9268 to 0.99167, *P* = 0.015, Fig. 3., Supplementary Table S1), ASA grade (OR = 0.3442, 95% CI: 0.1366 to 0.8667, *P* = 0.024, Fig. 3., Supplementary Table S1) and emergency indicator (OR = 2.4461, 95% CI: 1.59356 to 3.7546, *P* < 0.001, Fig. 3, Supplementary Table S1) were significantly associated with an increased risk of postoperative AKI.

Conversely, multiple variables were not significantly associated with postoperative AKI,.These included demographic factors (male gender, age), physiological parameters (height, weight), preoperative laboratory values (Hb, creatinine, uric acid), intraoperative measures (anesthesia duration, surgery duration, fluid intake/output, blood loss), hematological indices (PLT), hepatic function markers (ALT), surgical approach (laparoscopy, operation scale), and pre-existing medical conditions (pre-HTN, pre-CHD, pre-DM, pre-PVD, pre-HF). The lack of statistical significance for these variables (all *P* > 0.05, Fig. 3, Supplementary Table S1) suggests they may not serve as substantial predictors of postoperative AKI. These findings highlight the importance of NPAR and Alb as significant preoperative factors that can help predict the risk of postoperative AKI in patients undergoing non-cardiac surgery. The emergency indicator also emerges as a critical factor influencing postoperative AKI.

### Association of NPAR with postoperative AKI

A multivariate logistic regression analysis was performed to evaluate the predictive value of NPAR and its tertiles for postoperative AKI after performing a multicollinearity analysis. Variables with VIF > 10 were excluded (Supplementary Table S2). The unadjusted OR for NPAR was 1.094 (95% CI: 1.073–1.116, *P* < 0.001, Table 4), indicating a significant association with postoperative AKI risk. In Model 2, which adjusted for demographic and clinical characteristics including sex, weight, height, preoperative hypertension, preoperative coronary artery disease, preoperative diabetes mellitus, preoperative peripheral vascular disease, preoperative heart failure and age, the OR slightly decreased to 1.093 (95% CI: 1.072–1.116, *P* < 0.001, Table 4). In the fully adjusted Model 3, which further adjusted for intraoperative factors and comorbidities, such as emergency indicator, laparoscopy, volume of blood loss, anesthesia duration, volume of fluid intake, volume of fluid output, estimated 10-year survival rate, hemoglobin, uric acid and creatinine, the OR was 1.059 (95% CI: 1.034–1.084, *P* < 0.001, Table 4). For the middle tertile of NPAR, the risk progressively increased across models: 3.522 (95% CI: 1.840–6.742, *P* < 0.001, Table 4) in the unadjusted model, 3.538 (95% CI: 1.834–6.827, Table 4) in Model 2, and 2.633 (95% CI: 1.335–5.192, *P* = 0.00521, Table 4) in Model 3. The high tertile exhibited the strongest association, with ORs decreasing from 8.933 (95% CI: 4.873–16.377, *P* < 0.001, Table 4) in the unadjusted model to 4.403 (95% CI: 2.260–8.579, *P* < 0.001, Table 4) after full adjustment in Model 3. Notably, even after full adjustment in Model 3, the high NPAR tertile remained a significant predictor of postoperative AKI. A clear dose-response gradient was observed with adjusted ORs increasing from 2.633 (middle) to 4.403 (high tertile) in Model 3 (Table 4). The *P* for trend across all three models was less than 0.00001 (Table 4), which further corroborates the significant trend-like association between NPAR and the risk of postoperative AKI.

**Table 4:** Multivariate logistic regression analysis of AKI risk factors.

Exposure	Model 1 *P*-value	Model 2 *P*-value	Model 3 *P*-value
NPAR	1.094 (1.073, 1.116) < 0.001	1.093 (1.072, 1.116) < 0.001	1.059 (1.034, 1.084) < 0.001
Low NPAR tertile	1	1	1
Middle NPAR tertile	3.522 (1.840, 6.742) < 0.001	3.538 (1.834, 6.827) < 0.001	2.633 (1.335, 5.192) 0.00521
High NPAR tertile	8.933 (4.873, 16.377) < 0.001	8.819 (4.759, 16.343) < 0.001	4.403 (2.260, 8.579) < 0.001
*P* for trend	< 0.00001	< 0.00001	< 0.00001

Model 1: Not adjusted for other covariates.

Model 2: Adjusted for demographic and clinical characteristics including sex, weight, height, preoperative hypertension, preoperative coronary artery disease, preoperative diabetes mellitus, preoperative peripheral vascular disease, preoperative heart failure and age.

Model 3: Adjusted for Model 2 plus emergency indicator, laparoscopy, volume of blood loss, anesthesia duration, volume of fluid intake, volume of fluid output, estimated 10-year survival rate, hemoglobin, uric acid and creatinine.

## Discussion

In this study, we observed that the risk of postoperative AKI increased with elevated NPAR levels. This finding suggests that NPAR could serve as a novel biomarker for predicting the occurrence of postoperative AKI in non-cardiac surgical patients. The increase in NPAR reflects enhanced inflammatory responses, which is closely associated with AKI pathogenesis. Our findings align with the existing literature that identifies NPAR as a prognostic predictor in other diseases, further confirming its significance in inflammation-related conditions. The potential of NPAR as an independent predictor offers new insights into the early diagnosis and prevention of postoperative AKI, which may ultimately improve the clinical management of non-cardiac surgical patients.

Studies have demonstrated that inflammatory markers, including the NLR^28^, interleukin-18 (IL-18)^29^and tumor necrosis factor-α (TNF-α)^30^ are significantly elevated in patients with AKI. These markers not only indicate AKI severity but also predict prolonged hospitalization and increased mortality, underscoring their prognostic value^31,32^. Additionally, hypoalbuminemia is frequently observed in AKI patients and correlates with the severity of the condition, extended hospital stays, and higher mortality^33,34^. Hypoalbuminemia has been established as an independent prognostic factor for AKI, with lower albumin levels associated with poorer outcomes^35^. The anti-inflammatory and antioxidant properties of albumin may confer protective effects against AKI development, while reduced albumin levels reflect diminished capacity to counteract inflammation and oxidative stress^36^. As an emerging inflammatory marker, NPAR has recently demonstrated its potential clinical value in the study of various diseases^17,19,25^.

Our study found that patients with high NPAR levels exhibited significantly higher postoperative AKI incidence. Furthermore, we conducted a comparative analysis of NPAR against two key components of its calculation—neutrophil percentage (a marker of inflammatory activity) and albumin (a marker of nutritional status). ROC curve analysis demonstrated moderate predictive performance for NPAR (AUC = 0.723), which surpassed that of NLR (AUC = 0.705) and albumin alone (AUC = 0.685). This superior predictive capability likely stems from NPAR’s integration of two critical physiological dimensions: neutrophil-driven inflammatory activity (via neutrophil percentage) and systemic nutritional status (via albumin levels), thereby providing a more comprehensive assessment of inflammatory and metabolic derangements.

Emerging evidence highlights NPAR’s prognostic utility across multiple disease states. In patients with acute coronary syndrome, elevated NPAR predicts cardiovascular event risk (Hazard Ratio: 1.54, 95% CI 1.32–1.80)^37^ while in heart failure populations, it correlates with adverse outcomes, potentially reflecting cardiac inflammation and immune dysregulation^38^. Sepsis studies further demonstrate that increased NPAR levels associate with disease severity and mortality (AUC = 0.655)^39,40^, underscoring its role in evaluating infection-related systemic inflammation. Our study revealed significant intergroup differences in critical care utilization across NPAR tertiles (*P* < 0.001). Patients in the highest NPAR tertile (mean ± SD: 11.17 ± 6.74) exhibited substantially prolonged ICU stays (35.95 ± 141.55 days) and hospitalizations (22.75 ± 16.98 days) compared to middle (ICU: 10.90 ± 82.64; hospitalization: 18.82 ± 12.89) and low tertiles (ICU: 2.02 ± 25.85; hospitalization: 16.95 ± 8.00). The monotonic increase in care duration across ascending NPAR tertiles suggest a dose-response relationship between preoperative inflammatory-nutritional imbalance and postoperative recovery burden. Additionally, studies have demonstrated that NPAR serves as an independent predictor of contrast-associated acute kidney injury (CA-AKI) and long-term mortality in patients without chronic kidney disease (CKD) following percutaneous coronary intervention (PCI)^41^. NPAR is also linked to all-cause mortality in critically ill patients with AKI^42^. Our results extend these observations to postoperative AKI pathophysiology. Multivariable regression confirmed NPAR as an independent predictor of AKI, with ROC analysis demonstrating robust discriminative capacity (AUC = 0.723). Neutrophils are the primary effector cells in the inflammatory response, and an increase in their numbers indicates an enhanced inflammatory state. Concurrently, albumin levels decrease during this response, suggesting that elevated NPAR is typically associated with an intensified inflammatory response in the body. The rise in neutrophils can stimulate the release of inflammatory mediators and exacerbate oxidative stress^43^. Furthermore, the activation and aggregation of neutrophils may lead to microvascular obstruction^44^ thereby impacting the local hemodynamics of the kidney. Additionally, our findings demonstrate that NPAR not only serves as an independent predictor of postoperative AKI but also enables effective risk stratification in patients. Specifically, patients can be categorized into low-risk (NPAR < 4.07), intermediate-risk, and high-risk (NPAR ≥ 11.17) groups based on NPAR tertiles. The low-risk group exhibits a significantly lower incidence of postoperative AKI, whereas the high-risk group shows a markedly elevated incidence. Moreover, integrating NPAR with other inflammatory biomarkers (e.g., NLR) enhances predictive accuracy, thereby providing clinicians with actionable insights. Consequently, our findings suggest that monitoring preoperative and postoperative NPAR levels may be beneficial for patients undergoing non-cardiac surgery. Patients with persistently elevated preoperative NPAR levels accompanied by postoperative increases may require more intensive renal function surveillance and proactive clinical interventions. Future studies are necessary to further investigate these mechanisms and their implications for the role of NPAR in predicting and treating postoperative AKI.

In the current study, we demonstrated that NPAR exhibits moderate predictive performance (AUC = 0.723) for postoperative AKI, which surpasses the individual predictive power of neutrophil percentage (AUC = 0.705) and albumin (AUC = 0.685). While our analysis did not directly compare NPAR with established risk models such as the Revised Cardiac Risk Index (RCRI)^45^ or American Society of Anesthesiologists (ASA) physical status classification^46^ existing findings provide insights into potential complementary roles. The ASA grade, a widely used preoperative risk assessment tool, was identified as a significant independent predictor of postoperative AKI in our multivariable analysis (*P* = 0.024). Notably, the proportion of ASA grdade 3–5 patients increased progressively with higher NPAR tertiles, and their AKI incidence rose accordingly (from 1.18% in low NPAR to 9.66% in high NPAR). This suggests that ASA grade and NPAR may capture distinct aspects of risk: ASA grade reflects overall physiological status and comorbidity burden, while NPAR specifically quantifies the balance between systemic inflammation (neutrophil percentage) and nutritional status (albumin). Combining NPAR with established models like RCRI or ASA may enhance predictive accuracy. For example, RCRI focuses on cardiovascular risk factors, whereas NPAR reflects inflammatory and nutritional derangements—pathophysiological processes closely linked to AKI development. Integrating these complementary markers could provide a more comprehensive assessment of AKI risk, potentially improving stratification of high-risk patients. Future studies are warranted to directly compare NPAR with existing risk models and explore their combined utility, which may offer clinicians a more precise tool for preoperative AKI prediction and personalized management.

### Limitations

In our study, postoperative AKI was defined using the KDIGO creatinine-based criteria. It is important to note that urine output criteria were not incorporated into the definition of postoperative AKI. Urine output associated with postoperative fluid management and influenced by factors such as intravenous fluid administration and diuretic use. These factors can make urine output a less reliable indicator of renal function in the immediate postoperative period. Specifically, we acknowledge that the absence of urine output criteria in the postoperative AKI definition may limit the detection of some postoperative AKI cases that could be identified through combined creatinine and urine output criteria. Future studies may benefit from incorporating both creatinine and urine output criteria to provide a more comprehensive assessment of postoperative AKI.

We also recognize there are other limitations. First, baseline NPAR level varies with age, gender, ethnicity, and health status, potentially affecting its predictive reliability. Second, NPAR, being indicative of systemic inflammation, may be confounded by non-renal inflammatory conditions which complicates AKI prediction. Third, treatments such as antibiotics, anti-inflammatory medications, and blood purification therapies might also influence NPAR levels. Moreover, as this study is a secondary analysis of retrospectively collected data from a previously published dataset, the risk of selection bias and residual confounding cannot be fully eliminated. Although we adjusted for multiple covariates using multivariable regression, certain unmeasured inflammatory markers, such as interleukins and C-reactive protein may still have influenced the results. Finally, our data were derived from two tertiary hospitals in China, which may limit the generalizability of the findings to other populations or healthcare systems with different perioperative practices. Therefore, further validation through prospective, multicenter studies in more diverse populations is warranted to confirm the predictive utility of NPAR for postoperative AKI.

## Conclusion

Our findings suggest that NPAR may serve as a promising tool for risk stratification in patients undergoing non-cardiac surgery. As an accessible hematological marker, NPAR holds potential for early postoperative AKI diagnosis. Its changes over time could reflect treatment efficacy and inflammatory status, potentially guiding therapeutic decision-making. As a prognostic marker, NPAR may predict patient recovery and long-term survival rates. Integrating NPAR with other biomarkers and clinical parameters could enhance AKI prediction precision.

## Supplementary Information

Below is the link to the electronic supplementary material.

Supplementary Material 1

## Data Availability

Data Availability StatementThe data supporting the findings of this study are derived from the secondary analysis of a publicly available dataset. The original data were obtained from the study by Tang et al. (2022) and are openly accessible in the Dataverse repository under the persistent identifier DOI 10.1371/journal.pone.0270066. No new datasets were generated during the current study.

## References

[1] Zeuchner, J., Elander, L., Frisk, J. & Chew, M. S. Incidence and trajectories of subclinical and KDIGO-defined postoperative acute kidney injury in patients undergoing major abdominal surgery. *BJA Open.* **12**, 100345 (2024). 39483727 10.1016/j.bjao.2024.100345 PMC11526046

[2] Prowle, J. R. et al. Postoperative acute kidney injury in adult non-cardiac surgery: joint consensus report of the acute disease quality initiative and perioperative quality initiative. *Nat. Rev. Nephrol.* **20210511**, 605–618 (2021). 10.1038/s41581-021-00418-2 PMC8367817 33976395

[3] Deng, Y. et al. The incidence, risk factors and outcomes of postoperative acute kidney injury in neurosurgical critically ill patients. *Sci. Rep.* **7** (1), 4245 (2017). 28652590 10.1038/s41598-017-04627-3 PMC5484679

[4] Hu, L. et al. The incidence, risk factors and outcomes of acute kidney injury in critically ill patients undergoing emergency surgery: a prospective observational study. *BMC Nephrol.* **23** (1), 42 (2022). 35065624 10.1186/s12882-022-02675-0 PMC8782702

[5] Schiefer, J. et al. Incidence and outcomes of AKI in postoperative patients admitted to ICU using full KDIGO criteria - a cohort study. *J. Clin. Anesth.* **89**, 111156 (2023). 37356195 10.1016/j.jclinane.2023.111156

[6] Bonavia, A., Vece, G. & Karamchandani, K. Prerenal acute kidney injury-still a relevant term in modern clinical practice? *Nephrol. Dial Transpl.* **36** (9), 1570–1577 (2021). 10.1093/ndt/gfaa061 32596733

[7] Han, S. J. & Lee, H. T. Mechanisms and therapeutic targets of ischemic acute kidney injury. *Kidney Res. Clin. Pract.* **38** (4), 427–440 (2019). 31537053 10.23876/j.krcp.19.062 PMC6913588

[8] Patel, T. V., Kumar, S. & Singh, A. K. Post-renal acute renal failure. *Kidney Int.* **72** (7), 890–894 (2007). 17495862 10.1038/sj.ki.5002301

[9] Hamdi, A. et al. Severe post-renal acute kidney injury, post-obstructive diuresis and renal recovery. *BJU Int.* **110** (11 Pt C), E1027–E1034 (2012). 22583774 10.1111/j.1464-410X.2012.11193.x

[10] Matejovic, M. et al. Renal hemodynamics in AKI: in search of new treatment targets. *J. Am. Soc. Nephrol.* **27** (1), 49–58 (2016). 26510884 10.1681/ASN.2015030234 PMC4696587

[11] Devarajan, P. Pathogenesis of intrinsic acute kidney injury. *Curr. Opin. Pediatr.* **35** (2), 234–238 (2023). 36482770 10.1097/MOP.0000000000001215 PMC9992147

[12] Wang, Y. et al. Nanosystems for oxidative stress regulation in the anti-inflammatory therapy of acute kidney injury. *Front. Bioeng. Biotechnol.* **11**, 1120148 (2023). 36845189 10.3389/fbioe.2023.1120148 PMC9949729

[13] Li, Z. L. et al. Renal tubular epithelial cells response to injury in acute kidney injury. *Ebiomedicine* **107**, 105294 (2024). 39178744 10.1016/j.ebiom.2024.105294 PMC11388183

[14] Liu, W. et al. Regulation of renal ischemia-reperfusion injury and tubular epithelial cell ferroptosis by Ppargamma m6a methylation: mechanisms and therapeutic implications. *Biol. Direct*. **19** (1), 99 (2024). 39444036 10.1186/s13062-024-00515-9 PMC11515743

[15] Rabb, H. et al. Inflammation in AKI: current understanding, key questions, and knowledge gaps. *J. Am. Soc. Nephrol.* **27** (2), 371–379 (2016). 26561643 10.1681/ASN.2015030261 PMC4731128

[16] Sato, Y. & Yanagita, M. Immune cells and inflammation in AKI to CKD progression. *Am. J. Physiol. Ren. Physiol.* **315** (6), F1501–F12 (2018). 10.1152/ajprenal.00195.2018 30156114

[17] Zhu, Y. & Fu, Z. Association of Neutrophil-Percentage-To-Albumin Ratio(NPAR) with depression symptoms in U.S. Adults: a NHANES study from 2011 to 2018. *BMC Psychiatry*. **24** (1), 746 (2024). 39468499 10.1186/s12888-024-06178-0 PMC11520394

[18] Li, X., Gu, Z. & Gao, J. Elevated neutrophil percentage-to-albumin ratio predicts increased all-cause and cardiovascular mortality among individuals with diabetes. *Sci. Rep.* **14** (1), 27870 (2024). 39537759 10.1038/s41598-024-79355-6 PMC11560938

[19] Liu, C. F. & Chien, L. W. Predictive role of Neutrophil-Percentage-to-Albumin ratio (NPAR) in nonalcoholic fatty liver disease and advanced liver fibrosis in nondiabetic US adults: evidence from NHANES 2017–2018. *Nutrients* **15**, 8 (2023). 10.3390/nu15081892 PMC10141547 37111111

[20] Chen, Q. et al. Neutrophil percentage as a potential biomarker of acute kidney injury risk and Short-Term prognosis in patients with acute myocardial infarction in the elderly. *Clin. Interv Aging*. **19**, 503–515 (2024). 38525316 10.2147/CIA.S455588 PMC10959300

[21] Burn, G. L., Foti, A., Marsman, G., Patel, D. F. & Zychlinsky, A. *Neutrophil Immunity* **54**(7), 1377–1391 (2021). 10.1016/j.immuni.2021.06.006 34260886

[22] Kurkiewicz, K., Gasior, M. & Szygula-Jurkiewicz, B. E. Markers of malnutrition, inflammation, and tissue remodeling are associated with 1-year outcomes in patients with advanced heart failure. *Pol Arch. Intern. Med* **133**, 6 (2023). 10.20452/pamw.16411 36633195

[23] Ko, C. A. et al. Prognostic value of neutrophil percentage-to-albumin ratio in patients with oral cavity cancer. *Cancers (Basel)* **14**, 19 (2022). 10.3390/cancers14194892 PMC9564168 36230814

[24] Ji, W. et al. Association between neutrophil-percentage-to-albumin ratio (NPAR) and metabolic syndrome risk: insights from a large US population-based study. *Sci. Rep.* **14** (1), 26646 (2024). 39496695 10.1038/s41598-024-77802-y PMC11535182

[25] Li, J., Xiang, T., Chen, X. & Fu, P. Neutrophil-percentage-to-albumin ratio is associated with chronic kidney disease: evidence from NHANES 2009–2018. *PLoS One*. **19** (8), e0307466 (2024). 39102412 10.1371/journal.pone.0307466 PMC11299806

[26] Tang, Y. et al. Preoperative Neutrophil-Lymphocyte ratio for predicting surgery-related acute kidney injury in non-cardiac surgery patients under general anaesthesia: a retrospective cohort study. *PLoS One*. **17** (7), e0270066 (2022). 35905108 10.1371/journal.pone.0270066 PMC9337669

[27] Palevsky, P. M. et al. KDOQI US commentary on the 2012 KDIGO clinical practice guideline for acute kidney injury. *Am. J. Kidney Dis.* **61** (5), 649–672 (2013). 23499048 10.1053/j.ajkd.2013.02.349

[28] Zhou, X., Liu, J., Ji, X., Yang, X. & Duan, M. [Predictive value of inflammatory markers for acute kidney injury in sepsis patients: analysis of 753 cases in 7 years]. *Zhonghua Wei Zhong Bing Ji Jiu Yi Xue*. **30** (4), 346–350 (2018). 29663997 10.3760/cma.j.issn.2095-4352.2018.04.012

[29] Strauss, C., Booke, H., Forni, L. & Zarbock, A. Biomarkers of acute kidney injury: from discovery to the future of clinical practice. *J. Clin. Anesth.* **95**, 111458 (2024). 38581927 10.1016/j.jclinane.2024.111458

[30] McWilliam, S. J. et al. The complex interplay between kidney injury and inflammation. *Clin. Kidney J.* **14** (3), 780–788 (2021). 33777361 10.1093/ckj/sfaa164 PMC7986351

[31] Wei, W. et al. Neutrophil-to-Lymphocyte ratio as a prognostic marker of mortality and disease severity in septic acute kidney injury patients: a retrospective study. *Int. Immunopharmacol.* **116**, 109778 (2023). 36738677 10.1016/j.intimp.2023.109778

[32] Liu, Y. et al. Urinary Interleukin 18 for detection of acute kidney injury: a meta-analysis. *Am. J. Kidney Dis.* **62** (6), 1058–1067 (2013). 23830182 10.1053/j.ajkd.2013.05.014

[33] Zheng, L. J., Jiang, W., Pan, L. & Pan, J. Reduced serum albumin as a risk factor for poor prognosis in critically ill patients receiving renal replacement therapy. *BMC Nephrol.* **22** (1), 305 (2021). 34496793 10.1186/s12882-021-02512-w PMC8427850

[34] Yang, K. et al. The association between albumin and mortality in patients with acute kidney injury: a retrospective observational study. *BMC Nephrol.* **24** (1), 332 (2023). 37946135 10.1186/s12882-023-03323-x PMC10636863

[35] Xiang, F. et al. Expert consensus on the use of human serum albumin in adult cardiac surgery. *Chin. Med. J. (Engl)*. **136** (10), 1135–1143 (2023). 37083122 10.1097/CM9.0000000000002709 PMC10278724

[36] Wiedermann, C. J. & Joannidis, M. Nephroprotective potential of human albumin infusion: a narrative review. *Gastroenterol. Res.Pract.* **2015**, 912839 (2015). 10.1155/2015/912839 PMC4475554 26136776

[37] Liu, Y. et al. Associations between neutrophil-percentage-to-albumin ratio level and all-cause mortality and cardiovascular disease-cause mortality in general population: evidence from NHANES 1999–2010. *Front. Cardiovasc. Med.* **11**, 1393513 (2024). 39386385 10.3389/fcvm.2024.1393513 PMC11461234

[38] Wang, X. et al. The neutrophil percentage-to-albumin ratio is associated with all-cause mortality in patients with chronic heart failure. *BMC Cardiovasc. Disord*. **23** (1), 568 (2023). 37980510 10.1186/s12872-023-03472-9 PMC10657562

[39] Gong, Y., Li, D., Cheng, B., Ying, B. & Wang, B. Increased neutrophil percentage-to-albumin ratio is associated with all-cause mortality in patients with severe sepsis or septic shock. *Epidemiol. Infect.* **148**, e87 (2020). 32238212 10.1017/S0950268820000771 PMC7189348

[40] Hu, C. et al. Association between neutrophil percentage-to-albumin ratio and 28-day mortality in Chinese patients with sepsis. *J. Int. Med. Res.* **51** (6), 3000605231178512 (2023). 37314249 10.1177/03000605231178512 PMC10291015

[41] He, H. M. et al. Association between neutrophil percentage-to-albumin ratio and contrast-associated acute kidney injury in patients without chronic kidney disease undergoing percutaneous coronary intervention. *J. Cardiol.* **79** (2), 257–264 (2022). 34551865 10.1016/j.jjcc.2021.09.004

[42] Wang, B., Li, D., Cheng, B., Ying, B. & Gong, Y. The neutrophil percentage-to-albumin ratio is associated with all-cause mortality in critically ill patients with acute kidney injury. *Biomed Res Int* **2020**, 5687672 (2020). 10.1155/2020/5687672 PMC7049452 32219136

[43] Riaz, B. & Sohn, S. Neutrophils in inflammatory diseases: unraveling the impact of their derived molecules and heterogeneity. *Cells* **12**, 22 (2023). 10.3390/cells12222621 PMC10670008 37998356

[44] Perdomo, J. et al. Neutrophil activation and NETosis are the major drivers of thrombosis in heparin-induced thrombocytopenia. *Nat. Commun.* **10** (1), 1322 (2019). 30899022 10.1038/s41467-019-09160-7 PMC6428879

[45] Vernooij, L. M. et al. The comparative and added prognostic value of biomarkers to the revised cardiac risk index for preoperative prediction of major adverse cardiac events and all-cause mortality in patients who undergo noncardiac surgery. *Cochrane Database Syst. Rev.* **12** (12), CD013139 (2021). 34931303 10.1002/14651858.CD013139.pub2 PMC8689147

[46] Horvath, B., Kloesel, B., Todd, M. M., Cole, D. J. & Prielipp, R. C. The evolution, current value, and future of the American society of anesthesiologists physical status classification system. *Anesthesiology* **135** (5), 904–919 (2021). 34491303 10.1097/ALN.0000000000003947

